# Intravascular lithotripsy angioplasty for treatment of atherosclerotic coral-reef occlusion of the infrarenal aorta and its bifurcation

**DOI:** 10.1016/j.jvscit.2023.101106

**Published:** 2023-01-18

**Authors:** Michele Piazza, Francesco Squizzato, Chiara De Massari, Franco Grego, Michele Antonello

**Affiliations:** Division of Vascular and Endovascular Surgery, Department of Cardiac, Thoracic, Vascular Sciences and Public Health, School of Medicine, University of Padua, Padua, Italy

**Keywords:** Iliac artery, Intravascular lithotripsy, Peripheral arterial disease, Stents, Vascular calcification

## Abstract

In the present report, we have described the use of intravascular lithotripsy angioplasty for heavily calcified occlusions of the infrarenal aorta and its bifurcation in two patients. In the first patient, two lithotripsy balloons in kissing conformation were simultaneously used to allow for dilatation of the distal aorta and its bifurcation with preservation of accessory renal artery patency, followed by stenting of the iliac arteries. For the second patient, the infrarenal aorta occlusion was first treated with a single lithotripsy balloon, followed by covered stenting. Intravascular lithotripsy could represent a valid endovascular adjunct to optimize outcomes in the treatment of coral reef aortas and aortic bifurcation occlusion.

Severe aortoiliac obstructive disease, including type D Trans-Atlantic Inter-Society Consensus II lesions, has been traditionally treated with aortobifemoral bypass, although associated with considerable morbidity and mortality.^1^^,^^2^ More recently, endovascular treatment has emerged as a first-line approach for complex lesions involving the entire infrarenal aorta or its bifurcation.

Nevertheless, the presence of highly calcified vessels can represent a considerable challenge for endovascular repair, and the risk of life-threatening complications, such as arterial rupture or dissection,^3^, ^4^, ^5^, ^6^ must be considered. In addition, target lesion patency can be affected by the inability to achieve adequate intraoperative dilatation or lesion recoil. The use of covered stents can improve the patency rates. However, coverage of important side vessels and inadequate stent radial forces have remained problematic.

The Shockwave IVL (intravascular lithotripsy) device (Shockwave Medical, Inc, Santa Clara, CA) is a novel endovascular tool that uses an angioplasty balloon to deliver acoustic shockwaves to the arterial wall, creating microfractures in calcified plaques. The device is intended for the treatment of highly calcified lesions. By making the vessel more compliant, IVL can be used for vessel preparation or treatment, reducing the risk of wall rupture.^7^ It has been successfully used in the treatment of iliac obstructive disease. However, its application for disease extending into the aortic bifurcation and infrarenal aorta, especially in presence of complete occlusions, has not yet been described.

We have reported the case of two patients who had presented with extensive aortoiliac atherosclerotic disease characterized by heavily calcified occlusions involving the infrarenal aorta and its bifurcation. The patients provided written informed consent for the intervention and the report of their case details and imaging studies.

## Case report

### Patient 1

**Fig 1:**
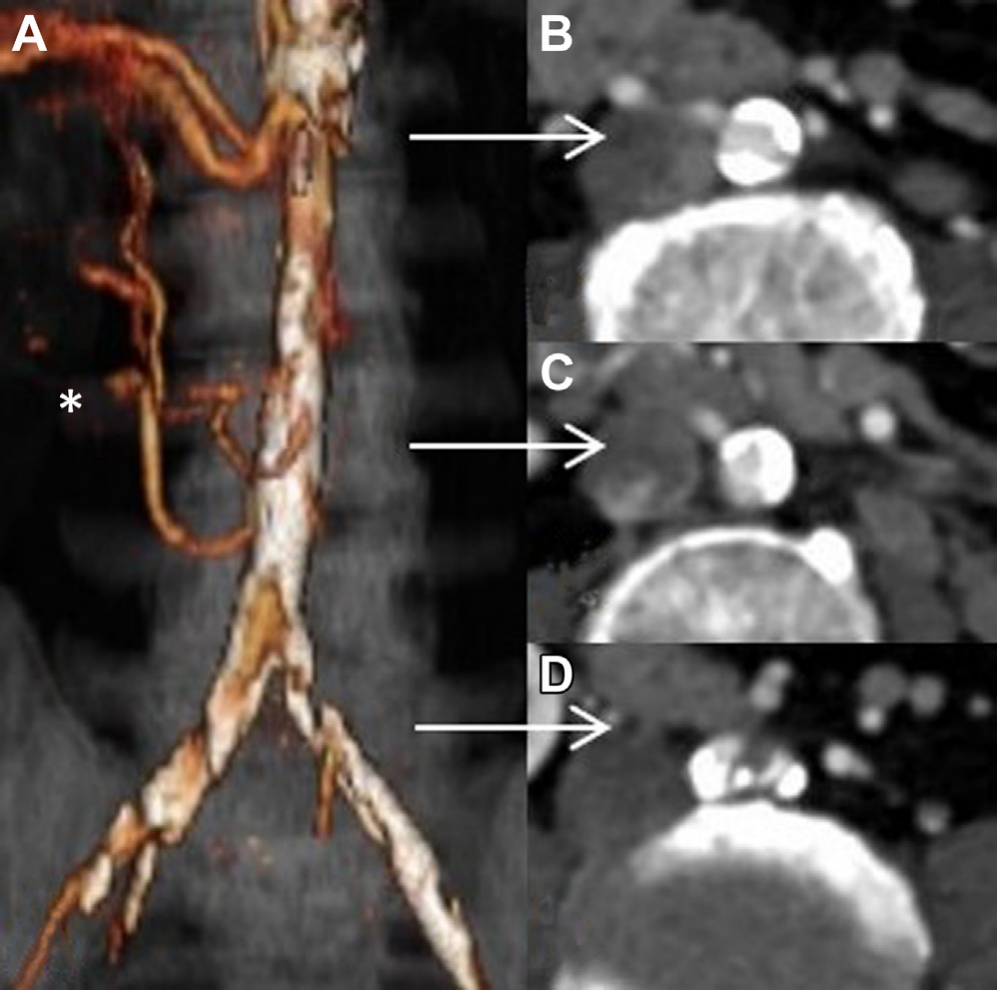
**A,** Three-dimensional reconstruction of preoperative computed tomography angiography (CTA) showing multiple renal arteries feeding a solitary right kidney (*asterisk*) and the maximum points of stenosis in the infrarenal aorta and its bifurcation (**B-D**).

A 52-year-old man had presented with bilateral lower limb rest pain, no palpable femoral pulses, and preoperative duplex ultrasound showing a bilateral femoral monophasic waveform. His medical history included smoking, hypertension, left nephrectomy, and laparoscopic partial resection of the right kidney. Computed tomography angiography (CTA) revealed extensive aortoiliac atherosclerotic lesions with heavily calcified occlusion of the aortic bifurcation and common iliac arteries. In addition, two large right accessory renal arteries originated from the distal aorta that was 9 mm in diameter, and the diameter of the iliac arteries was 6 mm (Fig 1).

**Fig 2:**
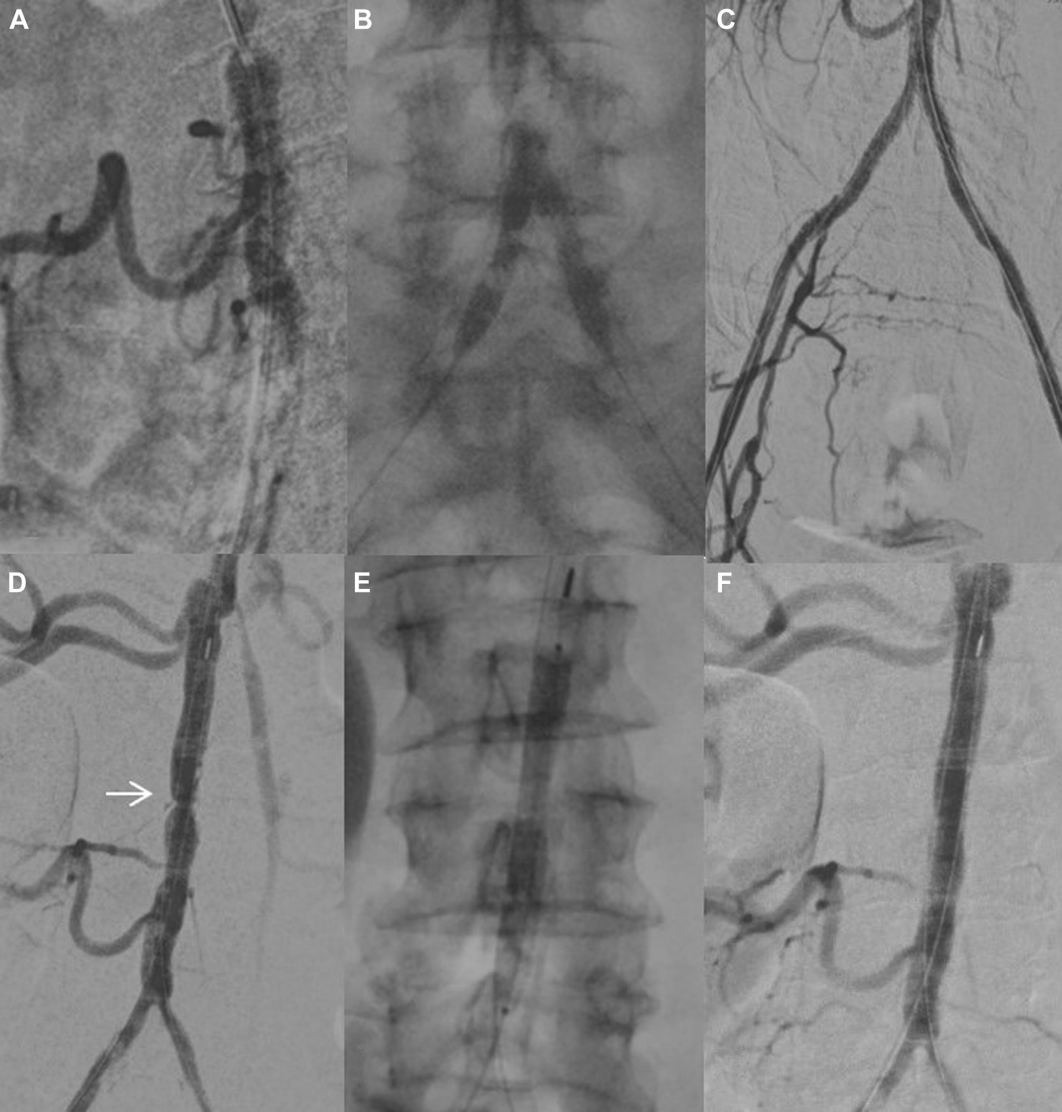
**A,** Intraoperative angiogram showing severe narrowing of the aorta below the main right renal artery. **B,** Two 6.5 × 60-mm Shockwave M5+ balloons positioned at the aortic bifurcation in a kissing conformation. **C,** Completion angiogram showing patency of the iliac axes and right internal iliac artery. **D,** Residual stenosis at the infrarenal aorta (*arrow*). **E,** An 8 × 60-mm Shockwave M5+ balloon positioned in the infrarenal aorta. **F,** Final angiogram showing satisfactory dilatation of the distal aorta, patency of the accessory renal arteries, and correct positioning of the iliac stents without signs of mutual compression or recoil.

After bilateral percutaneous femoral access and bilateral iliac recanalization, the aortoiliac bifurcation occlusions were predilated with a standard 5-mm balloon. Next, two 6.5 × 60-mm Shockwave M5+ balloons were positioned in the iliac axes, protruding into the distal aorta in a kissing conformation, each one attached to a dedicated generator. They were inflated to 6 atm repeatedly for 10 cycles to promote effective calcium fracture without the risk of contralateral plaque protrusion (Fig 2, *A-C*). An 8 × 60-mm Shockwave M5+ balloon was then advanced into the distal aorta and inflated for another 10 cycles, achieving satisfactory dilatation of the remaining infrarenal aortic stenosis (Fig 2, *D-F*). The procedure was completed by placement of bilateral iliac kissing stents, using two balloon-expandable covered stents (Lifestream, 6 × 38 mm; C.R. Bard, Providence, NJ), sparing the accessory renal arteries. Completion aortography confirmed patency of the distal aorta, right accessory renal arteries, and stented iliac axes, with no signs of stent compression. Postoperative duplex ultrasound showed a bilateral femoral triphasic waveform. At 12 months after the intervention, the patient had had no symptom recurrence, and CTA showed patency of the distal aorta, iliac axes, and right renal arteries.

### Patient 2

**Fig 3:**
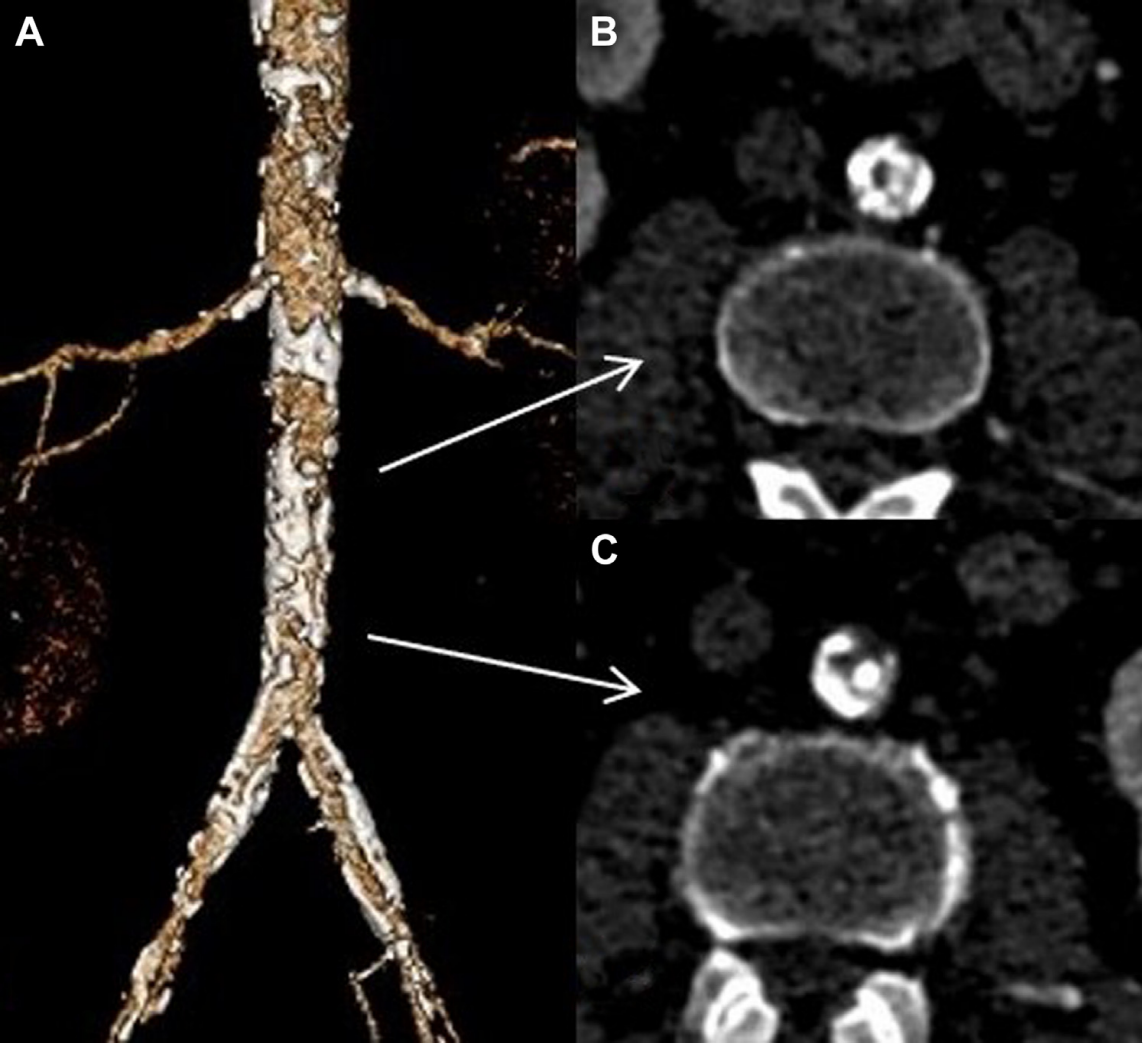
**A,** Three-dimensional reconstruction of preoperative computed tomography angiography (CTA) showing a “coral reef” aorta with occlusion of the mid-infrarenal aorta (**B**) and both common iliac arteries (**C**).

A 69-year-old man had been referred for life-limiting intermittent claudication that occurred after walking 65 ft, no palpable femoral pulses, and a monophasic waveform at both common femoral arteries. His medical history included hypertension, dyslipidemia, diabetes mellitus, and previous carotid endarterectomy. CTA revealed extensive aortoiliac obstructive disease with complete “coral reef” occlusion of the mid-infrarenal aorta and bilateral common iliac artery occlusion (Fig 3). The aortic diameter was 12 mm, and the diameter of the iliac arteries was 8 mm.

**Fig 4:**
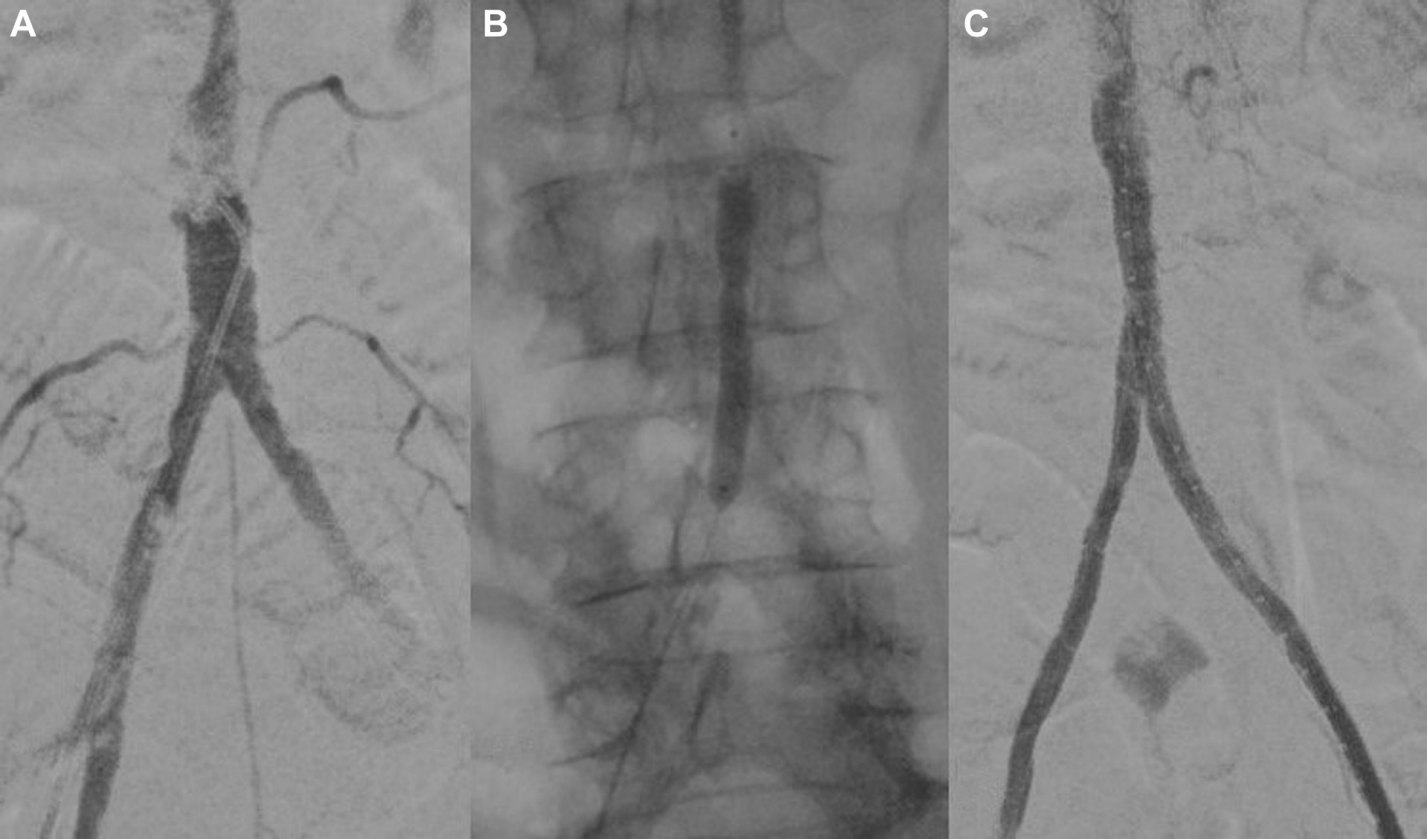
**A,** Intraoperative angiogram showing occlusion of the infrarenal aorta and multiple severe calcifications of the iliac axes. **B,** An 8 × 6-mm Shockwave M5+ balloon positioned in the distal aorta. **C,** Final angiogram showing satisfactory dilatation and patency of the treated aorta and iliac axes.

**Fig 5:**
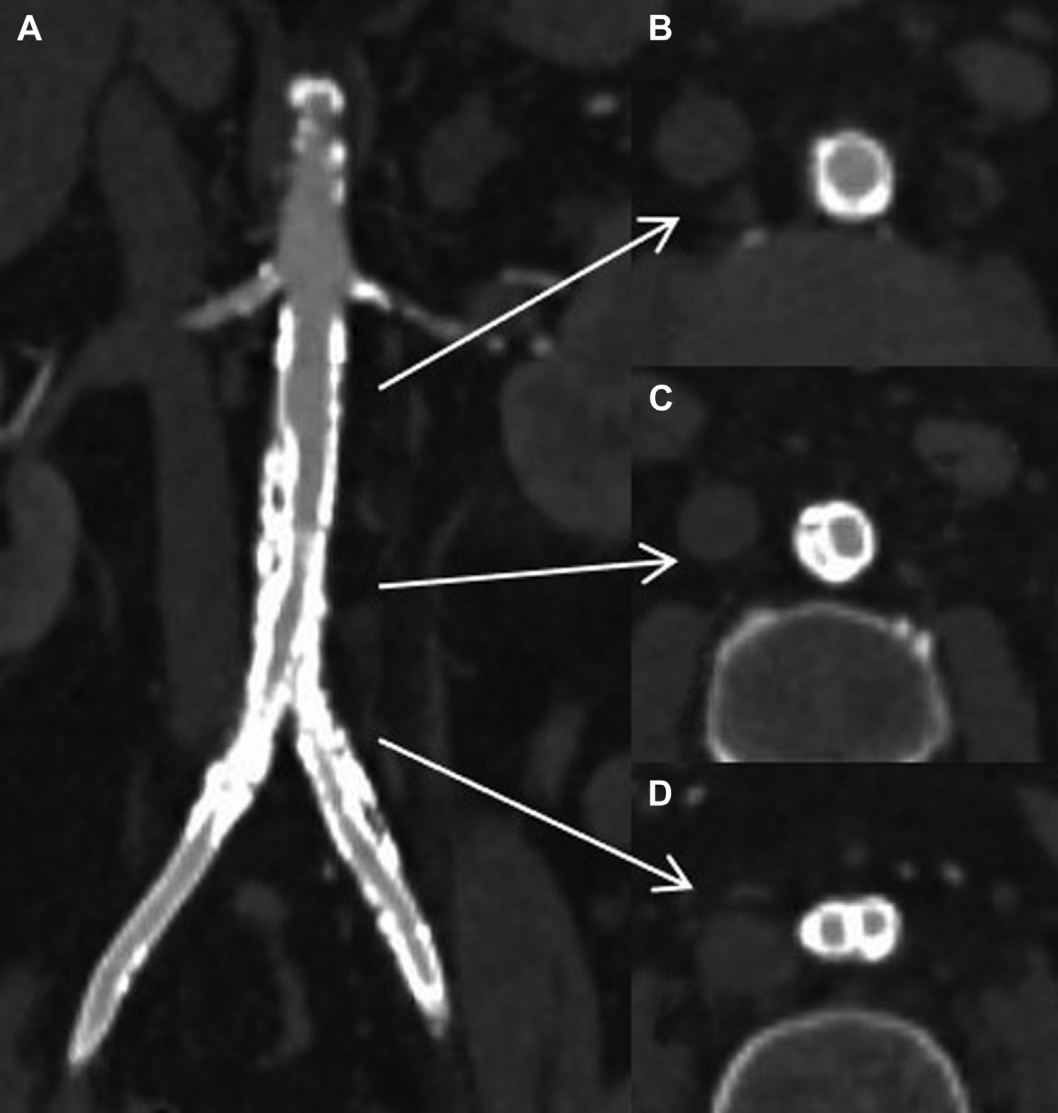
**A,** Reconstruction of postoperative computed tomography angiography (CTA) showing fracture of the calcified lesion at the mid-infrarenal aorta (**B)** and regular patency of the covered endovascular reconstruction of aortic bifurcation (**C-D**).

After bilateral percutaneous femoral access, the infrarenal aorta was predilated with a standard 5-mm balloon. Next, a 6.5 × 60-mm Shockwave M5+ balloon was advanced and progressively inflated to 6 atm during a 10-cycle period. An additional 10 cycles were delivered for aortic preparation using an 8 × 60-mm Shockwave M5+ balloon. To allow for adequate aortic dilatation from 8 mm to its 12-mm diameter, covered endovascular reconstruction of aortic bifurcation was performed, using an 11-mm Viabahn VBX stent (W.L. Gore & Associates, Flagstaff, AZ) expanded to 12 mm with a semicompliant balloon proximally.^8^ Completion aortography confirmed patency of the stented aorta and iliac axes, with no sign of stent recoiling or restenosis, and the duplex ultrasound scan showed a triphasic femoral waveform (Fig 4). At 12 months after the intervention, the patient had had no symptom recurrence. The postoperative CTA is shown in Fig 5.

## Discussion

Endovascular treatment of heavily calcified lesions of the abdominal aorta and its bifurcation has been hampered by the risk of arterial perforation or flow-limiting dissection, and the clinical outcomes have been affected by inadequate dilatation and/or early stent recoiling, leading to loss of patency. Thus, IVL might have an important role in providing effective calcium microfracture without the risk of rupture.^9^ IVL has been used for the treatment of coronary, iliac, and infrainguinal artery disease.^10^ However, the reported experience in the aortoiliac segment is limited. In the case of calcified iliac arteries, IVL has been used to provide safe vessel preparation before stent placement, with good procedural success and low rates of residual stenosis and complications.^11^ Also, the use of IVL, with or without associated stenting, has been proved useful to enable large-bore transfemoral access (ie, during transfemoral aortic valve implantation), with a reported reduction in access-related complications.^12^^,^^13^ To the best of our knowledge, we have described the first use of IVL for the treatment of the infrarenal aorta and aortic bifurcation. In our experience, in cases of total occlusion, predilatation with a plain angioplasty balloon might be required to allow for safe IVL balloon advancement, avoiding the risk of rupture. The use of two kissing IVL balloons, each one attached to a dedicated generator, could be advantageous for the treatment of the aortic bifurcation to avoid the risk of contralateral plaque shift during dilatation. The use of IVL by itself might be sufficient to achieve hemodynamic success and can be considered as a standalone treatment for selected cases. However, stabilization of the result through the deployment of covered stents might improve the durability.

In clinical practice, the use of IVL might be limited by its costs and availability, and, in our experience, its use has been limited to highly selected patients with heavily calcified aortoiliac obstructive lesions at risk of rupture during the procedure.

## Conclusions

IVL appears to be a safe and feasible option for the endovascular treatment of severely calcified occlusions of the infrarenal aorta and its bifurcation, with the theoretical advantage of avoiding the risk of rupture and preserving the patency of the side branches. Two kissing IVL balloons can be used for the treatment of the aortic bifurcation. Larger studies with longer follow-up are still necessary to investigate whether IVL alone can be sufficient to guarantee adequate patency over time or whether traditional stenting will still be necessary.
